# The Nurses' Tribute, Everybody's Business

**Published:** 1918-11-30

**Authors:** 


### AFTER THE ALLIES' TRIUMPHANT VICTORY.

### THE NURSES' TRIBUTE, EVERYBODY'S BUSINESS.

Now that the war is over, and the nation is rejoicing in triumphant victory, there is a danger that those who helped to win may be forgotten. **There is scarcely a home in Great Britain that does not owe a tribute to the British nurse.** I do not mean a tribute of praise, nor yet a halo! My theme is economics, in which sentiment, no doubt, occupies a place. But I am not a believer in sentiment that finds its finality in mere words. The Nurses' Tribute must be substantial and tangible—something that can be touched and handled, and it must be paid by the dwellers in the Homeland. No tribute will be more willingly or more generously paid, provided that the claim is adequately and promptly presented.

"SOME SWEETS FOR THE NURSES."

A year ago, or thereabouts, a friend of mine was summoned by War Office wire to France to see his son, a promising young officer, who was lying dangerously wounded in a hospital at Rouen. This youth had surrendered a valuable civil appointment to take his place in the fighting line, and he was surprised to see his father, a busy and practical man, for it had not occurred to him that his life was in danger. "Is there anything that you would like?" inquired the anxious parent. The boy smiled, and said, "Some sweets for the nurses, Dad." It was his last and only request. A few days later there was a military funeral, and to his credit be it said that when it was all over and done that gallant soldier's father entertained the nurses who had attended his son to a little dinner in a local hostelry.

BRITISH MEN AND WOMEN'S GIFTS WAITING.

I do not suggest that there is anything extraordinary or unique in this incident, and I mention it merely to illustrate my contention that not thousands, but millions, of British men and women are only waiting for an opportunity of discharging an obligation to the brave women who have spent themselves without stint on the wounded and broken to whose valour we owe the sweets of victory. The British nurse has helped to win the war, but she has done more than this. She has been the means of rescuing from the jaws of death a vast army of the victims of battle, many of whom are already restored, and others are on the high road to health. These nurses have not toiled for financial reward. They went forth like our men-at-arms to help to win the war, prompted only by love for their country and their kinsmen, and love for their work. They responded to the call of duty, and they went ready to share the fortunes of war, ready, if necessary, for death, and if the nation's debt is never paid they will not complain. To whine is not the British way.

"WANTED, A CHAMPION."

I am glad to notice advertisements in *The Times* from Lady Cowdray's Committee calling for response, and it is likewise gratifying to note that the Albert Hall Ball will bring in ten thousand pounds. But we want something more than this. We want a champion. We want a hundred champions. In the first place Parliament should be asked to vote a subsidy of thanksgiving to the nurse, and at the same time an appeal made to the nation for voluntary offerings, not by mere advertisements, but by such process of propaganda as will reach every home in England. We want a Nurses' Million. The national tribute is due not merely to those who toiled in the Red Cross hospitals at the battle-front. Thousands of nurses volunteered for service overseas, and had to be content to work in military, auxiliary, and civil hospitals at home, and although such duty as this is perhaps not so picturesque, it is equally valuable, as is abundantly testified to by the wounded who come to Blighty to recover or to die. Outside his immediate relatives there is nobody in England for whom Tommy has a warmer corner in his affections than "sister."

MAKE THE NATIONAL OBLIGATION PLAIN.

And what of the V.A.D.s? I have a suspicion that in certain select nursing circles the V.A.D.s' service is not altogether appreciated. The enemy has been sowing tares and making groundless suggestions that peradventure the V.A.D. after the war may pretend to being a trained nurse. I shall discuss this on a future occasion. In the meantime I desire to point out that the services of the V.A.D.s in this war were as valuable as they were indispensable, and that it would be despicably mean to deny the truth. I know of many large hospitals which have been successfully run by a matron, a few trained sisters, and a wholly untrained staff of V.A.D.s. To this nursing army the British people owe a debt they can never repay. It is impossible adequately for one person to compensate another who has saved his life, but it is not impossible to render thanks in substantial form, and in a form which will be useful and valuable. And, I repeat, the opportunity to do so must be given and the importance and urgency of the national obligation made plain.

NON PUTO!

It is desirable that the attitude of the Bedford Fenwick School towards the Nation's Tribute should be borne in mind. Not having inaugurated it, they denounce it as an "insult." In connection with the Irish Tribute, a certain sportsman started a Stock Exchange lottery which, like a great many other games of chance, was technically illegal. These people not only described this as "an impudent gamble" and "an insult," but they set the law in motion and had it stopped. Their action has cost the Irish Nurses' Tribute a thousand pounds. And then they pose as the nurse's true friend. *Non puto!* But there is no doubt that they did their work well—just as effectually as the Germans did when they murdered Edith Cavell.

WILL THE COLLEGE MISS THE TIDE?

The Right Way to make the nurse's cause triumphant is for the College to present her claim to the nation and to do so with boldness and without delay. It is a case of catching the tide or missing it. Every household will pay its tribute. The propagandist services of hospital committees should be enlisted. It is all in their interests that the nurse should be benefited. Similarly, the nurse herself should help the cause. Sound the loud timbrel in every city and hamlet in England—propaganda here, there, and everywhere. Why not a great Victory Ball in every Large City throughout the Homeland? Now is the time for a Victory Thanksgiving everywhere to the British Nurse. This is what is wanting to-day rather than vain academic discussions as to whether tweedledum or tweedledee shall triumph in the framing of an Act of Parliament. The nurse wants money, freedom, provision for age and infirmity. She wants holiday homes, cheap travelling—in a word, some of the sweets of life. Now is her moment and a grateful people's opportunity. Let everybody make a contribution in money or by remunerative personal service or both.

IERNE.

